# Retrospective analysis of mortalities in a tertiary care hospital in Northeast Ethiopia

**DOI:** 10.1186/1756-0500-7-46

**Published:** 2014-01-20

**Authors:** Asrat Agalu Abejew, Alemneh Smieneh Tamir, Mirkuzie Woldie Kerie

**Affiliations:** 1 Department of Pharmacy, Wollo University, Dessie, Ethiopia; 2 Department of nursing, Dessie Referral Hospital, Dessie, Ethiopia; 3 Department of Health Services Management, Jimma University, Jimma, Ethiopia

**Keywords:** Mortalities, Causes, Wards, Ethiopia

## Abstract

**Background:**

Mortalities in the health care set up are prevalent, and causes are multifactorial with variations from area to area and also from ward to ward in the same health care set up. Analysis of mortalities and its causes in Ethiopian hospitals including Dessie Referral Hospital is not adequately known. Thus, the aim of this study is to determine the prevalence of mortalities and its causes in the Dessie Referral Hospital, Northeast Ethiopia.

**Methods:**

A retrospective analysis of mortalities during a three year period (September 2010–2012) was conducted in the Dessie Referral Hospital from August-September, 2012. All in hospital mortalities in the hospital during the last three years were included in the study. Data were collected from patient discharge recording books. Finally, data were entered into SPSS windows version 16.0 and descriptive statistics were generated to meet the study objective.

**Results:**

During the last 3 years there were 1,481 (4.8%) mortalities in the hospital. Around 60.0% of the mortalities were among male patients, and two third of the mortalities were among patients aged 15 years or older. The majority of the mortalities (38.9%) were in the medical ward followed by pediatric (34.6%) and surgical (18.2%) wards. Most of the mortalities (34.8%) occurred during 2011 while least was in 2012 (31.8%). HIV/AIDS (14.8%), pneumonia (9.9%), and sepsis/shock (7.6%) were the three most common causes of mortality in the hospital during the three year period. On average, patients stayed for 2.86 (±2. 99) days in the wards before mortality.

**Conclusion:**

Mortalities in the wards of the Dessie Referral Hospital were high and the causes were mainly of infectious origin, HIV/AIDS and its complications being the most common causes. This calls for an integrated effort to reduce in hospital mortalities by equipping the hospital and its health care providers with the skills and medical supplies required for proper management of the most common causes of in hospital mortality reported in this study.

## Background

Health care settings are not as safe as they should be, due to mortality of the patient, which has traditionally been one of the most frequently used indicators of quality care. Mortality is the oldest known health care indicator and a valuable tool for planning and managing in hospitals [1,2]. The causes of mortality in hospitals are mostly human related problems [2] and disease conditions like infectious and non-infectious diseases [3]. Identifying the causes of mortality in hospitals is important for monitoring the health of the nations, identifying priorities, and national burden of disease analysis which attempts to estimate the causes of loss of healthy life [3].

The common causes of mortality in the health care set ups are infectious diseases (HIV/AIDS, Tuberculosis, and pneumonia) and non infectious (diabetes, stroke, ischemic heart disease, hypertensive heart diseases, chronic obstructive pulmonary disease, and cancer) [3-7]. The magnitude of mortality and causes are different from ward to ward even in one hospital [8-10]. The magnitude of mortality in the health care set up is more affected by state of hospitalization, length of stay, number of co-morbid conditions, and type of illness among others [6,8,11,12]. The majority of the causes are known to be preventable/avoidable [13] by devising preventable strategies such as guidelines and being compliant with it [9,13,14].

In Ethiopia the common causes of mortality were communicable diseases (59%), non-communicable diseases (31%) and injuries 12% [15,16]. This was similar with those reported in other countries [3-7]. The HIV/AIDs (11%) and tuberculosis (11%) were the common infectious causes of mortality in hospitals [15,16] whereas uterine rupture was responsible for 24% maternal mortalities [16,17].

Though different studies have been conducted in different parts of the world including Africa [8,12,14,18]. To our knowledge analysis of mortality in the general hospitals of the Ethiopia was not well studied except in particular wards and disease conditions [15-17]. This is particularly true in the study. Thus, this retrospective study was aimed to determine the frequency mortality and their causes in the tertiary care hospital in Ethiopia.

## Methods

### Study area

A retrospective descriptive study was conducted from August-September 2, 2012, in the Dessie Referral Hospital (DRH). The Hospital is located in Dessie town, Amhara Regional State in Northeast Ethiopia, 401km from Addis Ababa. It is a tertiary care hospital which is the only referral hospital in the region, and delivers a range of services including medical, surgical, gynecology and obstetrics, and pediatrics specialties. There are 16 governmental health institutions (1 referral hospital, 1 primary hospital, 8 health centers and 6 health posts), and 71 private health institutions (3 general hospitals, 6 higher clinics, 23 medium clinics, 15 pharmacies, and 24 drug stores) in the town.

### Study Subjects

All mortality registries from September 2010–2012 in the patient registry book of the hospital (started from implementation of the health management information system (HMIS)) were included in the study. To calculate the prevalence of mortality total admissions in each ward were included in the study.

### Data collection process

Data were collected using the structured format by nurses. The content of the format included demographic variables of patients on the patient record books (age, sex), the mortality and its causes. Total admissions were used to calculate rate of mortality in the hospital. Since this is a retrospective data correctness of diagnosis was not checked. Collected data were edited, coded, entered into SPSS windows version 16.0 and finally cleared and analyzed. Descriptive statistics were computed to meet the stated objectives.

### Ethical considerations

Ethical clearance was obtained from an ethical review committee of Wollo University. The management of the hospital was requested for cooperation/permission by a formal letter from the university. Names of physicians involved in the health care provision and those of patients were replaced by initials. All data obtained in the course of the study were kept confidential, and used solely for the purpose of the study.

## Results

During the last 3 years 1,481 (4.8%) mortalities occurred in the hospital. Majority 880 (59.2%) of the mortalities were among male patients, and among the age group less than 5 years 443 (29.4%). It is also worth to note that 1269 (85.0%) of the mortalities were among patients younger than 51 years old (Table 1).

**Table 1:** Sex and age distribution of mortalities during the last three years in the Dessie Referral Hospital, 2010–2012

**Patient characteristics**		**Frequency (%)**
***Sex***	Male	880 (59.4)
	Female	584 (39.4)
	Not mentioned	17 (1.2)
	**Total**	**1,481 (100)**
***Age***	Not mentioned	7 (0.5)
	<5 years	443 (29.4)
	5-14	70 (4.7)
	16-30	407 ( 27.5)
	31-50	343 (23.2)
	> = 51	212 (14.3)
	**Total**	**1,481 (100)**

Majority of the mortalities were in the medical ward 577 (38.9%) followed by pediatrics ward 513 (34.6%) and surgical ward 513 (34.6%). The magnitude of mortality declined over the three years in most of the wards though there was a slight increase in the Gynecology, pediatrics and Orthopedics wards (Table 2).

**Table 2:** Distribution of mortalities during the last three years in Dessie Referral Hospitals, 2010-2012

**Ward**	**Year**	**Total admissions**	**Mortality (%)**
Medical ward	2010	1, 417	190 (13.4)
	2011	1, 734	201 (11.6)
	2012	1, 753	186 (10.6)
	**Total**	**4, 904**	**577 (11.8)**
Pediatrics ward	2010	3, 666	179 (4.9)
	2011	3, 172	176 (5.6)
	2012	2, 407	158 (6.6)
	**Total**	**9, 245**	**513 (5.6)**
Surgical ward	2010	1, 571	97 (6.7)
	2011	1, 351	78 (5.8)
	2012	2, 561	94 (3.7)
	**Total**	**5, 483**	**269 (4.9)**
Gynecology	2010	1, 123	5 (0.5)
	2011	1, 208	8 (0.7)
	2012	1, 027	8 (0.8)
	**Total**	**3, 358**	**21 (0.6)**
Orthopedics	2010	456	8 (1.8)
	2011	362	6 (1.7)
	2012	378	8 (2.1)
	**Total**	**1, 196**	**22 (1.8)**
Obstetrics	2010	772	15 (1.9)
	2011	3, 129	47 (1.5)
	2012	2, 951	17 (0.6)
	**Total**	**6, 852**	**79 (1.2)**
**Total**		**31, 038**	**1, 481 (4.8)**

HIV/AIDS 219 (14.8%) was one of the common cause of mortality followed by pneumonia 146 (9.9%), sepsis/shock 113 (7.6%), and intestinal obstruction 103 (7.0%) (Table 3).

**Table 3:** The cause of mortalities in Dessie Referral Hospital, 2010-2012

**S. No.**	**Reasons for mortality**	**Frequency (%)**
1.	HIV/AIDS	219 (14.8)
2.	Pneumonia	146 (9.9)
3.	Sepsis/shock	113 (7.6)
4.	Intestinal obstruction	103 (7.0)
5.	Malnutrition	76 (5.1)
6.	Stroke	76 (5.1)
7.	Trauma and injury	71 (4.8)
8.	Preterm birth and low birth weight	68 (4.6)
9.	Road traffic accident	68 (4.6)
10.	Anemia	51 (3.4)
11.	Physical violence	51 (3.4)
12.	Congestive heart	47 (3.2)
13.	Meningitis	44 (2.9)
14.	Tuberculosis	38 (2.6)
15.	Liver disease	33 (2.2)
16.	Eclampsia	31 (2.1)
17.	Diabetic complications	28 (1.9)
18.	Uterine rupture	24 (1.6)
19.	Acute gastroenteritis	21 (1.4)
20.	Peptic ulcer disease and complications	21 (1.4)
21.	Poisoning	20 (1.4)
22.	Peptic ulcer disease and complications	19 (1.3)
23.	Post partum hemorrhage	16 (1.1)
24.	Malaria	14 (0.9 )
25.	Appendicitis and complications	13 (0.9)
26.	Renal disease	13 (0.9)
27.	Asthma and airway obstruction	12 (0.8)
28.	Burn	11 (0.7)
29.	Tetanus	10 (0.7)
30.	Cancer	6 (0.4)
31.	Others*	30 (2.0)
	**Total**	**1481 (100)**

Others*:*Myocardial infarction, abortion, acute febrile illness, antepartum hemorrhage, emphysema, hypertension, pharyngeal abscess, retained placenta, hernia, hemopneumothorax, gulian-Berry syndrome, deep venous thrombosis, croup, drug toxicity and anthrax, Asphyxia, Epilepsy, mechanism aspiration, hemopneumothorax, croup, Rabies.*

**Figure 1:**
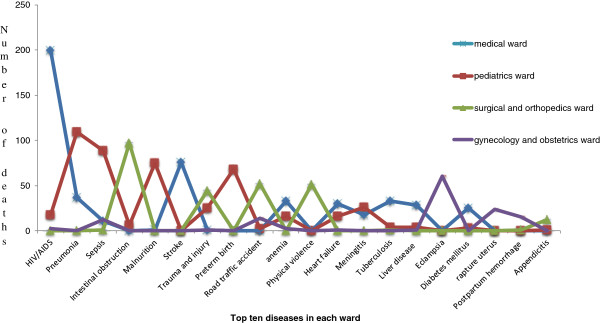
Top five diseases in each ward of Dessie Referral Hospital, 2010-2012.

When we consider ward specific causes of mortality, HIV/AIDS 199 (34.5%), stroke 76 (13.2%) and pneumonia 37 (6.4%) were the three most common causes in medical ward whereas pneumonia 109 (21.2%), sepsis 89 (17.3%) and malnutrition 75 (14.6%) were the three most common causes in the Pediatrics ward. On the other hand Eclampsia 30 (30.0%), ruptured uterus 24 (24.0%), and postpartum hemorrhage 15 (15.0%) were common causes in gynecology and obstetrics wards whereas intestinal obstruction 97 (33.3%), and physical violence 51 (17.5%) were the causes of mortality in the surgical and orthopedics wards (Figure 1).

Average length of stay of patients in the wards before mortality was 2.86 (±2. 99). The majority of patients 618 (41.7%) stayed only 1day in wards before mortality followed by 2 days 255 (17.2%) (Table 4).

**Table 4:** Distribution of mortalities in different wards in Dessie Referral Hospital, 2010-2012

**Variables**		**Ward**						**Total (%)**
		**Medical**	**Pediatrics**	**Surgical**	**Gynecology**	**Obstetric**	**Orthopedic**	
Year	2010	190	179	97	5	15	8	494 (33.4)
	2011	201	176	78	8	47	6	516 (34.8)
	2012	186	158	94	8	17	8	471 (31.8)
	**Total**	**577**	**513**	**269**	**21**	**79**	**22**	**1481 (100)**
Sex	Male	321	306	236	0	0	18	880 (59.5)
	Female	256	206	34	21	79	4	584 (39.5)
	**Total**	**577**	**513**	**269**	**21**	**79**	**22**	**1481 (100)**
Age	<5 years	0	442	1	0	0	0	443 (29.9)
	5-14	0	70	0	0	0	0	70 (4.7)
	15-30	218	0	103	16	58	11	406 (27.4)
	31-50	224	0	87	5	18	9	343 (23.2)
	>50 years	134	0	76	0	0	2	212 (14.3)
	Missed	1	1	2	0	3	0	7 (0.5)
	**Total**	**577**	**513**	**269**	**21**	**79**	**22**	**1481 (100)**
**Length of stay**	<1 day	8	7	5	0	0	1	21 (1.5)
	1 day	242	247	112	9	0	8	618 (45.3)
	2 days	119	93	37	1	0	5	255 (18.7)
	3 days	53	49	20	3	0	4	129 (9.2)
	4 days	37	34	16	3	0	0	90 (6.6)
	5 days	33	15	13	1	0	1	63 (4.6)
	6 days	15	16	12	0	0	1	44 (3.2)
	7 days	16	9	7	0	0	0	32 (2.3)
	8 days	10	6	9	1	0	1	27 (2.0)
	9 days	8	9	8	0	0	0	25 (1.8)
	1 days	2	4	4	1	0	0	11 (0.8)
	**≥11 days**	**25**	**7**	**13**	**2**	**0**	**0**	**47 (3.4)**
	Unspecified	9	18	13	0	79	1	120 (8.1)
	**Total**	**577**	**513**	**269**	**21**	**79**	**22**	**1481 (100)**

## Discussion

This study tried to analyze the mortalities from different wards of the referral hospital. The overall prevalence of mortality in the hospital was 4.8% out of the total admissions during the last three years. The prevalence was much lower when compared with study in Nigeria (11.1%) but relatively higher than two other studies in Greece (3.4%) and Sudan (3.7%) [8,19,20]. The ward specific mortality rates in this study were 11.8%, 5.6% and 4.9% in the medical ward, pediatrics ward and surgical ward respectively. This was relatively higher compared with ward specific mortality rates in Sudan [8]. This might be justified by differences in socio-cultural background of the population which affects health care seeking behavior and thus the outcome of admission.

In this study patient’s mean length of stay was 2.86 (±2. 99), and the majority (43.5%) of the mortalities occurred within twenty-four hours of admission. The pattern of mortality was relatively similar with findings reported in Sudan (59%) and Nigeria (>33%) [8,19]. This could be explained by the severity of the disease condition and late presentation of patients due to poor referral system, and lack of transportation and financial barriers. Since the figures are exhaustive (N = 1481) it could indicate that the peak mortalities occurred within 24 hours of admission in the hospital demonstrating the severity of disease conditions and relatively high patient flow in the hospital.

In this study, HIV/AIDS was the leading cause of mortality in the hospital accounting for 14.8% of total mortalities. This was similar with earlier studies in Ethiopia and elsewhere in Africa [3,15,19] which was 10.8%, 11.1%, and 12% in Ethiopia, Nigeria, and South Africa Respectively. This indicates that HIV/AIDS remains to be the major cause of mortality in African hospitals. In current study 5.1% of the mortalities are due to cerebrovascular diseases. This is not in line with other study in Spain (17.3%) and Nigeria (25%) [6,19]. However, it has to be noted that the cerebrovascular diseases are becoming important causes of mortality in Ethiopian hospitals as noted in this study unlike previous study in the country [15]. Pneumonia (9.9%), sepsis/shock (7.6%) and intestinal obstruction (7.0%) were the other leading causes of mortality in this study. This is similar to studies in Nigeria (pneumonia 12.8%, and septicemia 13.9%) and Spain (pneumonia 16.7%, and septicemia 13.6%) [6,21] except for intestinal obstruction. Hence, infectious diseases were the most common causes of mortality followed by cerebrovascular diseases and nutrition deficiencies. This was the case in other studies too [3,6,15,19,21].

Unlike other earlier studies road traffic accidents, physical violence and poisoning were among the common causes of mortalities in this study [3,6,19,21]. Road traffic accident was the most common cause of mortality in this study which is similar to previous study in Ethiopia (6%) [15]. But it was different from study in South Africa (1.0%) [3]. This difference might be due to under reporting of the mortalities from the traffic accidents [22] and the high traffic accidents in the Ethiopia is due to the geographic location and the night time driving practices exposing to accidents. The mortality from poisoning was higher than the one reported in other tertiary care hospital in Nigeria (0.5%) [21].

The causes of mortality were different from wad to ward in the hospital. Eclampsia 29.54%, ruptured uterus 18.18%, and post partum hemorrhage 13.64% were the most in Gynecology and obstetrics ward. This was relatively similar to a study in Ethiopia (eclampsia/ preeclampsia 35.7%, and ruptured uterus 12%), Nigeria (Eclampsia 14.6%, postpartum hemorrhage 13.3%, and ruptured uterus 8.0%), Bolivia (hemorrhage 13.3%, eclampsia 7.0%, & ruptured uterus 13.0%) and India (Eclampsia%, post partum hemorrhage 25%, and ruptured uterus 5.0%) [10,16-18,23-25]. This indicated that Eclampsia and post partum hemorrhage are global causes of maternal mortalities.

Being retrospective, this study faces so many challenges such as incomplete records, vagueness in recording and non-uniformity in record keeping across all wards. Due to this it wasn't possible for statistical analysis such as logistic regression and determine odds ratios to indicate if there is some association between age, sex and cause of mortality. Adverse drug reactions and medication errors were not included in the study. Another limitation of the study is that it may not accurately represent regional health statistics since it uses hospital data. Being retrospective, the diagnosis recorded in the patient registration book was taken as correct without verification.

## Conclusion

In conclusion, mortalities in the hospital were prevalent and the causes are multifactorial. HIV/AIDS and its complications are the most common causes of mortality followed by Pneumonia sepsis/shock, intestinal obstruction and stroke. Road traffic accident, physical violence and poisoning were preventable cause of mortality in the hospital. This calls for public health measures to reduce mortality by intervening on preventable causes. Prospective studies should also be initiated in the hospital, and in fact the hospital should design mortality reduction strategies. A concerted action by the health care team including all professionals (physicians, nurses, and clinical pharmacists) can greatly contribute to the reduction of mortality from treatable causes.

## Competing interests

The authors declare that they have no competing interests.

## Authors’ contributions

**AA** was involved in the design of the study, data analysis, and interpretation of the findings, report writing, review of the report, and manuscript preparation. **AS** was involved in the data analysis and interpretation of the findings, and writing and review of the report. **MW** was involved in the review of the final manuscript. All authors read and approved the final manuscript.

## References

[B1] Corrigan JM Donaldson MS Kohn LT Institution of medicine: shaping the future for health. To err is human: building a safer health system 1999 Washington, DC: National academy press IOM document repository, last accessed on 15/11/ 2010. URL:/ http://www.iom.edu 25077248

[B2] Ben-Tovim D Woodman R Harrison JE Pointer S Hakendorf P Hen-ley G Measuring and reporting mortality in hospital patients 2009 Can-berra: Australian: Institute of Health and Welfare Cat. No. HSE 69

[B3] Bradshaw D Pillay-Van WV Laubscher R Nojilana B Groenewald P Nannan N Metcalf C Cause of death statistics for South Africa: Challenges and possibilities for improvement Burden of Disease Research Unit 2010

[B4] Zwi K Pettifor J Soderlund N Meyers T HIV infection and in-hospital mortality at an academic hospital in South Africa Arch Dis Child 2000 83 227 230 10.1136/adc.83.3.227 10952640 PMC1718469

[B5] Roaeid RB Kablan AA Diabetes mortality and causes of death in Benghazi: a 5-year retrospective analysis of death certificates EMHJ 2010 16 1 65 69 20214160

[B6] Caballero PP García JJC Ojesto AG Arrieta NR-G Fernández CS Galán CDA Early mortality in a tertiary care hospital: analysis of quality of care Emergencias 2011 23 430 436

[B7] Leblan PA Impact of HIV/AIDS on Trends in Major Causes of Death at a Rural Mission Hospital in Kenya: Review of 4858 Records Annals of African Medicine 2006 5 3 142 148

[B8] Ayrton J Attwood D Kuron LD A Retrospective Analysis of Mortality Distribution in Juba Teaching Hospital, Southern Sudan SSMJ 2008 2 1 last accessed on 15/11/ 2012. URL:/http://www.southernsudanmedicaljournal.com

[B9] Malomo IO Aina OF Ladapo HTO Owoeye AO Ten-year mortality review in a pioneer psychiatric hospital in West Africa East African Medical Journal 2003 80 7 379 383 16167755 10.4314/eamj.v80i7.8723

[B10] Oladapo OT ALamina M Fakoya TA Maternal deaths in Sagamu in the new millennium: a facility-based retrospective analysis BMC Pregnancy and Child birth 2006 6 6 1 7 10.1186/1471-2393-6-6 PMC1434770 16529649

[B11] Freemantle N Weekend hospitalization and additional risk of death: An analysis of inpatient data J R Soc Med 2012 1 11 22307037 10.1258/jrsm.2012.120009 PMC3284293

[B12] Walsh B Roberts HC Nicholl PG Features and outcomes of unplanned hospital admissions of older people due to ill-defined (R-coded) conditions: Retrospective analysis of hospital admissions data in England BMC Geriatrics 2011 11 62 1 7 22011327 10.1186/1471-2318-11-62 PMC3209437

[B13] Boman H Bjo¨rnstig U Hedelin A Eriksson A “Avoidable” Deaths in Two Areas of Sweden – Analysis of Deaths in Hospital after Injury Eur J Surg 1999 165 828 833 10.1080/11024159950189294 10533755

[B14] Bachou H Tumwine JK Mwadime RKN Tylleskär T Risk factors in hospital deaths in severely malnourished children in Kampala, Uganda BMC Pediatrics 2006 6 7 1 9 16542415 10.1186/1471-2431-6-7 PMC1472687

[B15] Misganaw A HaileMariam D Araya T Ayele K Patterns of mortality in public and private hospitals of Addis Ababa, Ethiopia BMC Public Health 2012 12 1007 10.1186/1471-2458-12-1007 23167315 PMC3520706

[B16] Abdella A Maternal Mortality Trend in Ethiopia Ethiop.J.Health Dev 2010 24 1 115 122

[B17] Gessessew A Melese MM Ruptured uterus-eight year retrospective analysis of causes and management outcome in Adigrat Hospital, Tigray Region, Ethiopia Ethiop.J.Health Dev 2002 16 3 241 245

[B18] Marivate M Towobola O Theron E Stefan V Maternal and perinatal mortality figures in 249 South African hospitals 1988–1992 S Afr Med J 1996 86 4 409 412

[B19] Fadare JO Afolabi AO The pattern of medical mortalities in a specialist hospital in North-central Nigeria Annals of Ibadan Postgraduate Medicine 2010 8 2 101 105 25161476 10.4314/aipm.v8i2.71824 PMC4111025

[B20] Papadopoulos IN Papaefthymiou M Roumeliotis L Panagopoulos VG Stefanidou A Kostaki A Status and perspectives of hospital mortality in a public urban Hellenic hospital, based on a five-year review BMC Public Health 2008 23 8 28 10.1186/1471-2458-8-28 PMC2257958 18215322

[B21] Adeolu AA Arowolo OA Alatise OI Osasan SA Bisiriyu LA Omoniyi EO Odesanmi WO Pattern of death in a Nigerian teaching hospital; 3-decade analysis African Health Sciences 2010 10 3 266 272 21327138 PMC3035961

[B22] Samuel JC Sankhulani E Qureshi JS Baloyi P Thupi C Lee CN Miller WV Cairns BA Charles AG Under-Reporting of Road Traffic Mortality in Developing Countries: Application of a Capture-Recapture Statistical Model to Refine Mortality Estimates PLoS ONE 2012 7 2 e31091 doi:10.1371/journal.pone.0031091 10.1371/journal.pone.0031091 22355338 PMC3280223

[B23] Olopade FE Lawoyin TO Maternal Mortality in a Nigerian Maternity Hospital African Journal of Biomedical Research 2008 11 267 273

[B24] Jain M Maharahaje S Maternal Mortality-A retrospective analysis of ten years in a tertiary hospital Indian J.prev.soc.med 2003 34 3&4 104 111

[B25] Ro¨o¨st M Altamirano VC Liljestrand J Esse´n B Priorities in emergency obstetric care in Bolivia––maternal mortality and near-miss morbidity in metropolitan La Paz BJOG 2009 116 1210 1217 10.1111/j.1471-0528.2009.02209.x 19459864

